# Remote Speech and Language Therapy services in Buckinghamshire

**Published:** 2012-06-15

**Authors:** Adam Willison, Debbie Begent

**Affiliations:** Buckinghamshire County Council, UK; Buckinghamshire County Council, UK

**Keywords:** telehealth, speech and language therapy, joint commissioning

## Abstract

**Executive summary:**

Speech and Language Therapists work with people with aphasia following Stroke. For many clients aphasia is a life-long condition and an individual’s ability to adjust to this is very variable. Buckinghamshire Healthcare Trust has an integrated adult Speech and Language Therapy (SLT) Service with 3.6 WTE therapists allocated to the Community Long-Term Conditions Team which serves patients with aphasia as well as other conditions including dementia and progressive neurological conditions. Obviously there is a limit in the capacity of this service and we need to look to alternative and innovative methods of service provision. These have included:

- group therapy
- strengthen links with the voluntary sector
- Conversation Partner Scheme training volunteers to visit people in their own homes

**Strategic context:**

A local healthcare need was identified by Buckinghamshire County Council (BCC); difficulty accessing intensive SLT for clients with aphasia post stroke. BCC had experience and expertise in Telecare/Telehealth and wanted to extend the role of Telehealth to these clients, funding was identified. BCC and the SLT Department worked together for several months identifying the best quality package for people with aphasia. This was potentially very exciting but the initial concept required adjustment as the current Telecare was not sophisticated enough to cope with the data produced by complex aphasia computer therapy. An additional partner was brought in ‘Steps Consultancy’ and this enabled us to produce a package which achieved our initial objective. The provision of the following is secure for 3 years: 7 MSI touchscreen computers (with keyboard and mouse). Three laptops and laptop bags. Multiple user Licence for Step-By-Step software. Licence x8 REACT software. Ten finger scanners, microphones and headphones. Contract with a local company for hardware support. Continued software support form Steps including staff training.

**Case for change:**

Currently we are set up to begin using the devices remotely, this took a lot of technical work with Steps Consultancy and Buckinghamshire Healthcare Trust (BHT) IT Department. SLT staff was trained in software use but are not yet highly familiar with the package. All devices were assigned to clients and we are in the process of setting these clients up with a system by connecting the device to the central server through their home internet system. The outdated IT equipment we are trying to use within the Trust is slowing us down. Also, due to staffing constraints in the department the assessment of patients, setting up of the programme, delivering and setting up the equipment and the following up on results and updating therapy packages is time consuming and has been difficult to absorb into our current capacity, but we are making progress.

**Objective:**

To provide people with aphasia access to Teletherapy so that they can increase the amount of therapy practice.

**Service Proposal for stage 2:**

The SLT department has identified 3.75 hours a week of therapy time to dedicate to the Teletherapy Project, this will be subject to BHT approval, if approved will commence January 2012. This is in order to use the equipment to its full potential and achieve timely distribution of computer therapy packages. We have concerns that, as all touchscreens are allocated to patients, and patients keep the screens for approximately 6 months there is going to be a waiting list for equipment as we receive new referrals for Teletherapy. The SLT Department proposes further development of the project in the following ways:

- investment in upgrading the project to teleconferencing to use the equipment we currently have to its full potential. This would involve investment in Broadband, new up to date Windows7 PCs and 24” monitors (3 of each), webcams and appropriate software to allow teleconferencing;
- purchasing a further 10 touchscreen computers so that the programme is available to new strokes without having to go on a waiting list;
- increased consultancy fee to Steps so that we can have mentorship of our new post-holder who will have 3.75 hours per week allocated to the project;
- to provide SLT services to other neuro-conditions that result in a SLT requirement;
- integration of telehealth service into existing community equipment services and countywide Telehealth monitoring contract to test model for Telehealth at scale in next 12–24 months.

